# Traditional Herbal Medicine Candidates as Complementary Treatments for COVID-19: A Review of Their Mechanisms, Pros and Cons

**DOI:** 10.1155/2020/2560645

**Published:** 2020-10-10

**Authors:** Rhea Veda Nugraha, Hastono Ridwansyah, Mohammad Ghozali, Astrid Feinisa Khairani, Nur Atik

**Affiliations:** ^1^Graduate School of Biomedical Sciences Master Program, Faculty of Medicine, Padjadjaran University, Bandung, Indonesia; ^2^Department of Biomedical Sciences, Faculty of Medicine, Padjadjaran University, Bandung, Indonesia; ^3^Immunology Study Group, Faculty of Medicine, Padjadjaran University, Bandung, Indonesia

## Abstract

Coronavirus disease 2019 (COVID-19) is a new infectious disease caused by severe acute respiratory syndrome coronavirus 2 (SARS-CoV-2) that belongs to the coronavirus family. The first case was reported in December 2019, and the disease has become a pandemic. Impaired immune regulation is one of the factors that play a role in its pathogenesis and results in poor outcomes of COVID-19 patients. There have been many studies with drug candidates used as antivirals or immunomodulators. However, the results of these investigations showed that the drug candidates were not significantly effective against the disease. Meanwhile, people believe that consuming herbal immunomodulators can prevent or even cure COVID-19. Unfortunately, specific preclinical and clinical trials to evaluate the effects of herbal immunoregulators have not been conducted. Certain natural compounds might be effective for the treatment of COVID-19 based on general concepts from previous experiments. This review discusses some herbal agents extracted from various plants, including *Echinacea*, *Cinchona*, *Curcuma longa*, and *Curcuma xanthorrhiza*, which are considered for the treatment of COVID-19. In addition, we discuss the pros and cons of utilising herbal medicine during the COVID-19 pandemic, draw some conclusions, and make recommendations at the end of the session.

## 1. Introduction

The current pandemic of COVID-19 that is spreading across countries originated in Wuhan, China [1]. The single cause of this highly communicable disease is a novel coronavirus, called severe acute respiratory syndrome coronavirus 2 (SARS-CoV-2), which is the seventh known virus of the Coronaviridae family capable of infecting humans [2]. The latest report from the World Health Organization cited that there are now over 19 million confirmed cases and over 700,000 deaths worldwide caused by this virus. The United States of America now has the highest number of COVID-19 cases (over 4 million cases), followed by Brazil (almost 3 million cases) and India (over 2 million cases) [3]. The fast propagation of this disease is mainly through close contact with infected individuals via respiratory droplets from either sneezing or coughing. Furthermore, there are two other ways of transmitting the virus, including contact and aerosol transmission [4].

Among infected patients, COVID-19 shows various unspecific symptoms, ranging from mild to severe. A report from Huang et al. mentioned that fever (98%) is the most frequent manifestation that is reported by patients, followed by cough (76%), myalgia or fatigue (44%), sputum production (28%), and headache (8%) [5]. Also, some fatal cases have been reported in certain patients experiencing progressive respiratory failure due to the virus activity that attacks the alveolar epithelial cells. This damage is initiated by the receptor-binding domain (RBD) attachment of the virus to the receptor on the respiratory tract, known as the angiotensin-converting enzyme-2 (ACE2) receptor [6]. Humans have many ACE2 receptors in their respiratory tracts, which increase their susceptibility to COVID-19. This molecular mechanism may partly explain why the incidence rate of this disease is increasing rapidly. Afterwards, viruses infecting humans can lead to subsequent inflammatory processes and the release of numerous proinflammatory cytokines that are responsible for the clinical appearance of inflammation. Some of these proinflammatory cytokines, including IL-2, IL-7, IL-10, G-CSF, IP-10, MCP-1, MIP-1a, and TNF-*α*, are highly elevated in the blood of severely ill COVID-19 patients. Thus, there may be an association between this elevated level of cytokines and the severity of a patient's manifestations [5].

Currently, there is no specific treatment for COVID-19. Furthermore, people in the community and researchers are trying to find the best way to cure or prevent the disease, including using herbal medicine. Since the immune status of patients plays an essential role in COVID-19 infection, an herbal medicine, which has an immunomodulatory effect, could have potential as a preventive measure and even therapeutic agent for patients with COVID-19 infection [7, 8]. A recent trend in the community is the consumption of herbal medicines containing certain active compounds, which have antimicrobial or antiviral, anti-inflammatory, and immunostimulatory activities, such as echinacea, quinine, and curcumin. These herbal compounds are assumed to have the capacity to modulate the immune response and, therefore, they are believed to have beneficial effects on preventing or treating COVID-19 [8, 9].

*Curcuma longa*, for example, has been used traditionally by many countries in Asia as a drug or supplement because of its antioxidant, anti-inflammatory, antimutagenic, anticancer, and antimicrobial effects [9]. Many branded products contain an active compound that can act as an antiviral and immunostimulator. During the spread of COVID-19, several pharmaceutical companies offered their mainstay herbal products commercially. Consequently, based on the information from the advertisements on television, radio, and online media, people competitively shopped for these herbal products to fight against COVID-19. Unfortunately, this community behaviour was undertaken without a solid research base on the herbal effects of COVID-19. Studies that describe the relation of some herbal drugs with the molecular mechanisms of COVID-19 infection, treatment, and prevention remain to be explained.

This review explains in detail the molecular pathomechanism of COVID-19 and how active compounds of herbal products might prevent or cure the disease. It also will discuss the safety aspects of these natural compounds and provide recommendations for both the community and clinicians.

## 2. Characteristic of SARS-CoV-2

### 2.1. Structure of SARS-CoV-2

SARS-CoV-2, or severe acute respiratory syndrome coronavirus 2, is one of the viruses that belong to coronaviruses [10]. The common coronavirus is a positive single-strand RNA virus (+ssRNA) that belongs to the order Nidovirales, family Coronaviridae, and subfamily Orthocoronavirinae. This specific coronavirus is divided into four genera: *α*, *β*, *γ*, and *δ*. Each genus is further divided based on the characteristics of its subtype, genome, and phylogenic clustering. At present, six types of coronavirus infect humans. Thus, SARS-CoV-2 has become the seventh of coronavirus that can infect humans. SARS-CoV and the Middle East respiratory syndrome coronavirus (MERS-CoV) are coronaviruses that infect humans. Both SARS-CoV and MERS-CoV belong to the *β* genus that is a predecessor for the SARS-CoV-2. The other types of human coronavirus are 229E and NL63 that belong to the *α* genus and also OC43 and HKU1 that belong to the *β* genus [11, 12].

This novel SARS-CoV-2 has a ∼30 kb long RNA, which is the same length found in SARS-CoV and MERS-CoV that caused problem several years ago. This novel virus has a genome-sequence similarity of 96% and 79.5%, respectively, with a known bat coronavirus and SARS-CoV [12, 13]. Another study indicated that this virus had a genome-sequence similarity of about 45%–90% with SARS-CoV and a lower similarity of about 20%–60% for MERS-CoV [11]. Taken together, this SARS-CoV-2 has a less distant genomic sequence with a known bat coronavirus and SARS-CoV, but it has more distance with MERS-CoV.

Coronavirus is a type of virus that has an envelope, which is round or oval and is often pleomorphic. The diameter of this virus is about 50–200 nm [10–12]. SARS-CoV-2 has a unique, club-shaped spike projection on the surface of the virus, which makes this virus look like a solar corona [10, 12]. There are four major structural proteins encoded by this novel coronavirus. They are the spike (S), membrane (M), envelope (E), and nucleocapsid (N) proteins. The S protein is the most important structure that can bind with the receptor. This glycoprotein is located on the surface of the virus and mediates the attachment to the host cell's receptor [6, 12]. This protein is also the main antigenic structure in this virus [11].

The S protein of this virus is a trimeric S glycoprotein, which is a class I fusion protein and mediates the attachment to the host cell's receptor. In most coronaviruses, the S protein is cleaved by a host cell furin-like protease into two polypeptides, which are known as S1 and S2. The S1 is part of a large RBD of the S protein, and the S2 forms the stalk of the spike protein structure [12].

The S protein of SARS-CoV-2 can interact strongly with the host cell via the ACE2 receptor. This interaction creates a significant public health risk for human transmission [10, 11]. After the S protein of the virus binds with ACE2, the virus envelope can fuse with the host cell membrane and enter the host cell [6, 10].

### 2.2. Internalisation Process of the Host Cell of SARS-CoV2

SARS-CoV2 needs a specific receptor, the ACE2 receptor, to enter the host cell. The internalisation process of this novel virus initiates the interaction between the spike protein of the virus and the ACE2 receptor of the host cell [6, 10]. The initiation process begins with the interaction between the RBD within the S1 region of the virus S protein with the ACE2 receptor. After this interaction, the virus must have access to the host cell cytosol, which is accomplished by acid-dependent proteolytic cleavage of spike protein by a cathepsin or another protease. Then, the internalisation process begins with the fusion of the coronavirus and the membrane of the host cells. The spike protein cleavage occurs at two sites within the S2 portion. The first cleavage is essential to separate the RBD and fusion domains of the S protein. The other is to expose the fusion peptide or cleave at S2 [12].

**Figure 1:**
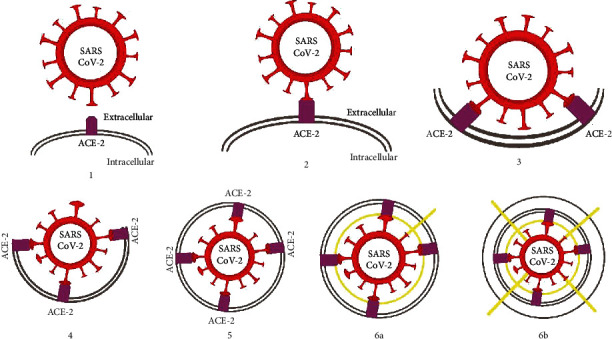
Internalisation process SARS-CoV-2 with its receptor. Red is a spike protein (epitope) of SARS-CoV-2, blue is the ACE2 receptor, and yellow is the serine protease. It shows the internalisation process of this virus into the host cell has occurred (6a and 6b).

The fusion between the virus and host cell usually occurs within acidified endosomes. Some coronaviruses also can fuse at the plasma membrane. The cleavage exposes the fusion peptide that inserts into the membrane at S2. This process is followed by the joining of two heptad repeats in S2 to form an antiparallel six-helix bundle. Bundle formation leads to the mixing of viral and host cell membranes and results in the fusion and release of the viral genome into the cytoplasm (Figure 1) [12].

### 2.3. Immune Reaction after Infection

The initial site of SARS-CoV-2 infection remains unknown, and the pathogenesis of COVID-19 is under investigation. In most patients with COVID-19, it may affect only the lung, because this disease is a respiratory disease. However, in some patients with certain comorbidities, the clinical symptoms might be worse. The mode of infection is human to human transmission through close contact. Close contact increases the risk of transmission via droplets, such as from an infected person through coughing or sneezing or the interaction between health workers and patients with COVID-19. This disease has an incubation period of about 2–14 days, and during this time, the virus can be transmitted. Thus, this mode of infection makes the rate of spread of this disease ranges from 2.2 to 2.6, which means one infected person can infect from 2.2 to 2.6 people [14].

Limited information is available about innate immune status against SARS-CoV-2 in infected patients. A report showed that COVID-19 patients had an increase in neutrophils, interleukin (IL-6), and C-reactive protein (CRP) and a decrease in lymphocytes. The innate immune system against viruses depends on how the interferon (IFN) type 1 response works effectively. This IFN and its downstream effects might regulate replication of the virus and induction of the adaptive immune response [14]. This shows how the innate immune system works in COVID-19.

Serology information about SARS-CoV-2 also remains unclear. Most patients with COVID-19 showed a peak increase of immunoglobulin M (IgM) nine days after disease onset and shifted to immunoglobulin G (IgG) in the second week [14]. Other data showed that viral load increases mostly during the first week of the disease but decreases in the second week. Then, IgM and IgG begin to increase on day 10 [15].

Recently, in some patients, several viruses might have caused an emerging situation because of an immune reaction. This emerging threat is known as cytokine release syndrome or a cytokine storm. This condition happens after infection with some influenza viruses [16]. It might also occur in infections caused by SARS-CoV-2. A study provided evidence to suggest that a subgroup of patients with severe COVID-19 might have cytokine release syndrome (CRS) or a cytokine storm. A cytokine storm became the second leading cause of death of COVID-19 because of an underrecognised and hyperinflammatory syndrome that leads to hypercytokinaemia with multiorgan failure [17].

Cytokine storm syndrome (CSS), also known as cytokine storm, is a condition in which there are an aggressive proinflammatory response and lack of control of an anti-inflammatory response after viral infection. These may happen depending on the severity of the disease and are the result of the interaction between viral virulence and host resistance. CSS begins with the site of infection that is the choreographer of cytokine amplification during infection, particularly in respiratory epithelial cells. After the virus enters the host cell, it will replicate within these cells and infect other cells, including alveolar macrophages. An immune response is triggered by apoptosis or necrosis of the infected cells. This immune response activates proinflammatory cytokines. Then, these cytokines lead to the recruitment of inflammatory cells. This process results in immune cell infiltration and tissue damage. The regenerative process also happens when tissue damage occurs. It will cause serious changes in organs, and their cytokines can enter the circulation where they become a cytokine storm, which leads to multiorgan failure [16].

In COVID-19, this cytokine storm is characterised by the increase of interleukin (IL)-2, IL-7, granulocyte-colony stimulating factor, IFN-*γ*, inducible protein (IP)-10, monocyte chemoattractant protein (MCP)-1, macrophage inflammatory protein (MIP) 1-*α*, and tumour necrosis factor (TNF)-*α* [17]. Another study showed that, in the CSS condition, there are increasing levels of IL-6, IL-8, and MCP-1 [14, 17]. Taken together, in the CSS condition, there are increasing levels of some proinflammatory cytokines.

The laboratory findings mentioned above are in line with SARS and MERS. The lymphopenia and cytokine storm may play an essential role in the pathogenesis of COVID-19. This cytokine storm may become an initial process of viral sepsis and inflammation-induced lung organ damage. This process leads to another complication, including pneumonitis, acute respiratory distress syndrome, respiratory failure, multiorgan failure, and potentially death [14].

## 3. Available Therapy for Managing COVID-19

A definitive therapeutic agent for managing COVID-19 has not been recommended for humans until now. Current preventive and treatment efforts for COVID-19 have focused developing vaccines and specific therapeutic agents targeting SARS-CoV-2 [18].

The preventive method that could potentially hamper the spread of diseases is vaccination. A vaccine for COVID-19 involves the active immunisation of the vaccine components to induce the production of neutralising antibodies specific to the SARS-CoV-2 antigen. The target antigen of the antibody is the S protein found on the surface of the virus. Some experimental studies have shown that giving the full-length S protein may induce the release of a protective antibody by blocking the binding of the virus with the ACE2 receptor [19]. Clinical testing of this vaccine is being conducted by some investigators from the National Institute of Allergy and Infectious Diseases-Vaccine Research Center (NIAID-VRC) [18].

There are some potential therapeutic agents for COVID-19 management, such as antiviral agents, chloroquine/hydroxychloroquine, dexamethasone, and convalescent plasma transfusion, but most of them still show inconsistent results. Several antiviral agents are under investigation as a treatment for COVID-19. Remdesivir is one of the antiviral agents that used for COVID-19 and known as an adenosine analogue that can be merged into viral RNA chains resulting in their early termination [20]. Davies et al. studied about the systematic benefit-risk assessment of remdesivir in the treatment of COVID-19. This study showed that there might be a favourable benefit-risk profile for remdesivir compared with placebo in severe cases of COVID-19 infection. There is still a need for benefit and safety data for remdesivir to provide further studies [21]. Wang et al. reported that remdesivir exhibited a promising effect *in vitro* [20]. However, a randomised, double blind, placebo-controlled, multicentre clinical trial of remdesivir was stopped early because of serious adverse events among the treatment and control groups. They also reported that the results were not associated with a difference in time to clinical improvement. Moreover, the further investigation suggested that remdesivir did not significantly reduce the mortality risk of the patients (8%) compared with the placebo group (11.6%), and NIAID ended the trial because of the insufficient benefit results and unethical issues [22–24].

Verdugo-Paiva et al. conducted a systematic review of the use of lopinavir/ritonavir for treatment of COVID-19. Lopinavir/ritonavir is a fixed-dose combination antiviral that is used to treat HIV infection. This antiviral known as an inhibitor of HIV protease enzyme formed an inhibitor-enzyme complex. This complex could prevent the cleavage of gag-pol polyproteins. In *in vitro* study, the agents had antiviral activity against SARS-CoV and MERS-CoV infections. In clinical study, the use of lopinavir/ritonavir reduced the intubation rate, steroid requirements, and mortality in SARS patients. This study reported that the use of lopinavir/ritonavir for COVID-19 might reduce mortality, risk of developing respiratory failure, acute respiratory distress syndrome, or requiring invasive mechanical ventilation, but the certainty of this evidence is low [25]. Other researchers conducted a randomized controlled trial and showed that lopinavir/ritonavir did not significantly reduce in viral clearance in both nonsevere and severe cases of COVID-19. These studies also suggested using lopinavir/ritonavir associated with some adverse events, mainly in the gastrointestinal tract [26, 27].

Other treatments that had been investigated are chloroquine and hydroxychloroquine. Chloroquine can enhance the endosomal pH needed by the virus to fuse with the host's cell membrane. This mechanism results in the inhibition of viral infection *in vitro* [20]. However, Gautret et al. reported in an open-label nonrandomised clinical trial that the combination of hydroxychloroquine and azithromycin is efficient in clearing nasopharyngeal viral carriage of SARS-CoV-2 in COVID-19 patients in three to six days [28]. But, Cortegiani et al. suggested in their systematic review that there has not been high-grade quality randomized controlled trial which can prove the efficacy and safety of chloroquine/hydroxychloroquine. Furthermore, the combination of chloroquine/hydroxychloroquine and macrolide showed a harmful event during treatment and might not reduce the rate of COVID-19 infection [29].

Because severe cases of COVID-19 are often associated with cytokine release syndrome, the use of corticosteroids may reduce the proinflammatory cytokines involved. Singh et al. in their RECOVERY trial study about the role of corticosteroid for COVID-19 showed a significant reduction in death with dexamethasone in severe cases on ventilator or moderate cases on supplemental oxygen therapy. This study also indicated that there was no effect of using dexamethasone in mild to moderate cases requiring no oxygen therapy and only gave benefit in severe cases. Nevertheless, to confirm the reliability, a multicentre study is necessary [30].

Rajendran et al. in their systematic review about convalescent plasma transfusion (CPT) for the treatment of COVID-19 suggested that CPT could be an effective therapeutic option with promising evidence on safety, improvement of clinical symptoms, and reduced mortality, in addition to antiviral/antimicrobial drugs. Besides, CPT also reduces the viral load and increases the level of antibody over time significantly. CPT was also well tolerated by all of participants in the studies. However, these data were only obtained from case reports and series. There has not been a high-quality randomized controlled trial to establish the efficacy and safety [31].

## 4. Herbal Medicine Candidates

In this situation, in which the preventive and therapeutic agents have not been established and recommended for administration to patients, herbal medicines are frequently used by many people in the community. According to the characteristics of the SARS-CoV-2 virus, a molecular mechanism of the host is involved in the immune response. As discussed above, we did a critical review of several papers from different journals. We highlighted at least four herbal medicines that could prevent or supplement the treatment of COVID-19 patients. In addition, we discuss these four candidates, including the pros and cons of their effects.

### 4.1. *Echinacea purpurea*

*Echinacea purpurea* (*E. purpurea*) is one of the most popular herbal medicines in Europe and North America because it shows promising effects against viral infections. Its common name is *Purple coneflower*. The preparation of *E. purpurea* can be made in the form of extracts, tinctures, teas, and sprays. Many Native Americans use this kind of herb for respiratory infections [8, 32]. It contains several bioactive compounds like chicoric acid and caffeic acids, alkylamides, and polysaccharides [33].

The utilisation of *E. purpurea* extracts for the treatment of virus-induced diseases expanded in the community after some studies reported that it had advantages as antiviral activity. In contrast, whether it has either anti-inflammatory or immunostimulatory effects remain debatable. Sharma et al. mentioned that echinacea extract was an interesting antivirus herbal treatment against some viruses with a membrane through direct virucidal activity. Viruses with membranes are likely very sensitive to echinacea administration, with herpes simplex virus at MIC100 0.39 *μ*g/mL, influenza 0.58 ± 0.22 *μ*g/mL, and RSV 2.5 *μ*g/mL. In contrast, in nonmembrane viruses, the MIC100 level is 800 *μ*g/mL in rhinoviruses and is higher in adenoviruses. Based on this report, since the human coronavirus is an enveloped virus, it may be a promising target for the effect of *E. purpurea* that can lead to a treatment for COVID-19 [8].

Some commercial products containing echinacea claim that they have an immunomodulatory effect and act mainly as an immunostimulator rather than an immunosuppressor. This claim is supported by a study in the USA, by Burger et al. They demonstrated that echinacea mediated the increased release of various cytokines, including IL-1, IL-10, and TNF-*α* by macrophages [34]. Macrophages may have a minimal number or no ACE2 receptors on their surface. They are considered to have the constant capability for the phagocytosis of SARS-CoV-2 and release TNF-*α* with other proinflammatory cytokines through the humoral immune response [14]. Under certain conditions, these cytokines can help to support immune cell activities, yet they may sometimes lead to various unwanted effects on tissues. TNF-*α* is a central cytokine that plays an important role in local tissue inflammation, such as an increase in vascular permeability, adhesion, and migration of leukocytes and secretion of other cytokines including IL-1 and IL-6 by activated leukocytes [35, 36]. It can be considered when a series of cytokines are liberated in COVID-19 with the supplementation of echinacea. So, it certainly will increase the number of proinflammatory cytokines induced by echinacea.

**Figure 2:**
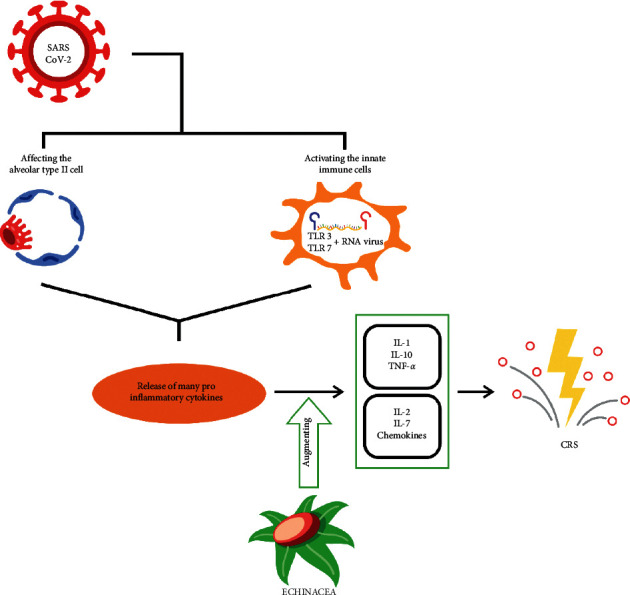
Proposed mechanism in which echinacea can enhance the production of IL-1, IL-10, and TNF-*α* in COVID-19 that can lead to cytokine release syndrome.

This adverse event can render hypercytokinaemia, then known as CRS. In mild cases, the liberation of cytokines only shows mild-to-moderate symptoms, such as fever, local harm, or the possibility of hypotension that can be adequately treated by fluid and vasopressor administration. However, if this vicious circle continuously happens, CRS may lead to a life-threatening condition, including shock and the necessity of mechanical ventilation [37]. A study indicated that COVID-19 patients treated in the intensive care unit with severe clinical features had a high level of innate cytokines IL-2, IL-10, IL-7, TNF-*α*, chemokines IP-10, MCP-1, and MIP-1A. Based on this report, it may be considered that the severity of COVID-19 symptoms is associated with a cytokine storm condition (Figure 2) [5].

Unlike the previous studies, Sharma et al. suggested that echinacea has an anti-inflammatory effect by inhibiting the production of proinflammatory cytokines induced by the virus, such as IL-6, IL-8, and TNF-*α*. It can be analysed that, after echinacea administration to a group of virus-infected cells, cytokine productions are inhibited, as in cultured cells with RV1A infection. A cytokine level in “infected cells without echinacea” group was at 625 ± 25 pg/mL for IL-6 and 2269 ± 38.5 pg/mL for IL-8, and in “infected cells with echinacea” group was at 36.2 ± 1.2 pg/mL (IL-6) and 196.9 ± 3.8 pg/mL (IL-8) [8]. This report exhibits that the immunomodulatory effect of echinacea is an immunosuppressor rather than an immunostimulator because of the inhibitory activity of various cytokine secretions.

No clinical trial is available to present evidence on the efficacy and safety profiles of echinacea administration to COVID-19 patients. The *in vitro* study showed a beneficial effect of echinacea as a virucidal agent. However, whether it has harmful effects must be further investigated in a clinical setting.

### 4.2. Curcumin

Turmeric is an herbal plant called rhizomatous and is also known as *Curcuma longa* (*C. longa*). It is a plant that has received much interest in the medical and scientific fields. Turmeric also belongs to the ginger (*Zingiberaceae*) family and the *Curcuma* genus [38]. Turmeric is mostly used as an important spice, a natural food colouring, and as food flavour. For many years, turmeric has been widely known as a medicinal plant to treat various diseases and conditions. The treatment with turmeric has been investigated in preclinical and clinical studies [9, 38].

*C. longa* has been used traditionally in Asian countries as a medicine or supplement. This medicine serves as an antioxidant, anti-inflammatory, anticancer agent and is used in the treatment for many other diseases, such as diabetes mellitus, cardiovascular disease, obesity, neurodegenerative disease, inflammatory bowel disease, allergy or asthma, and psoriasis [9, 38]. *C. longa* contains carbohydrate (96.4%), protein (6.3%), fat (5.1%), mineral (3.5%), and moisture (13.1%). On the other hand, its extracts contain curcuminoids, which are curcumin (77%), demethoxycurcumin (DMC; 17%), and bisdemethoxycurcumin (BDMC; 3%). All these curcuminoids act medicines or supplements, especially curcumin [9].

The most common kind of curcuminoid that is used as a medication is curcumin. Some other studies demonstrated that curcumin could be used as a treatment for hypertension [39, 40]. On the other hand, a systematic review suggested that the use of curcumin as an antihypertensive needs a longer time to have its effect on lowering blood pressure, more than 12 weeks approximately [39]. Some studies about the effect of curcumin demonstrated that ethyl acetate extracts of turmeric could decrease blood pressure with an angiotensin-converting enzyme inhibitor [40–42].

**Figure 3:**
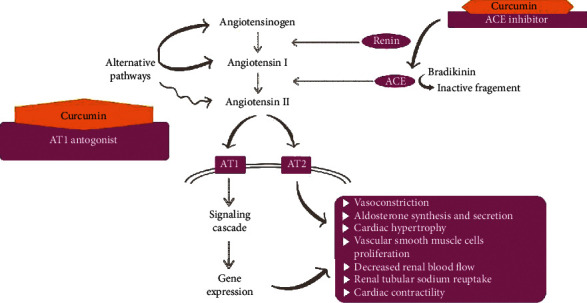
Curcumin's role in blood pressure as an antihypertensive agent, ACE inhibitor, and AT1 blocker that can inhibit the AT1 receptor in the renin-angiotensin-aldosterone system (RAAS).

Another study indicated that curcumin's mechanism of action for lowering blood pressure was through decreasing angiotensin II receptor type 1 (AT1). This mechanism prevented angiotensin II from binding with AT1 or decreased the number of angiotensin II that could bind with AT1. Blocking the binding process resulted in the inability of the blood vessel to constrict, a decrease in renal and another tissue perfusion, and resulted in a reduction in blood pressure (Figure 3) [43].

An earlier study showed that one of the most common comorbidities in COVID-19 patients is hypertension (23.7%). In hypertension, there is an increase of angiotensin-converting enzyme (ACE). Thus, one of the antihypertension agents has a mechanism that inhibits ACE and is called an ACE inhibitor [44]. However, the administration of an ACE inhibitor activates a feedback mechanism that can increase the amount of ACE in the body to maintain homeostasis. Curcumin activates the same mechanism when it is administered as an ACE inhibitor [42, 44, 45].

An investigation in 2005 showed that ACE2 was not inhibited by the administration of ACE inhibitors in hypertensive patients [46]. A new study suggested an interplay between COVID-19 and the renin-angiotensin-aldosterone system (RAAS). A preclinical study demonstrated that an RAAS inhibitor such as ACE inhibitor or angiotensin-receptor blocker (ARB) might increase ACE2 expression. On the other hand, another study indicated that the administration of an RAAS inhibitor did not influence the ACE2 receptor [47]. Taken together, we concluded that the results are inconsistent regarding the administration of the RAAS inhibitor since they may or may not increase the ACE2 receptor.

Based on these data, there is a possibility that the administration of curcumin to prevent COVID-19 might cause a highly susceptible individual to get infected with SARS-CoV-2 and eventually become worse. This process occurs because the novel virus has a specific receptor, which is ACE2, that is also involved in the hypertension mechanism itself. Administration of curcumin, as a RAAS inhibitory agent, may increase the expression and production of ACE2. Therefore, the use of curcumin for prevention might cause susceptible people to become infected with COVID-19. Thus, curcumin as a treatment for COVID-19 should be reconsidered, because it causes an increase in ACE2 that might worsen the progression of this disease. A clinical trial study is needed to confirm the effect of using curcumin as a preventive agent against COVID-19.

On the other hand, curcumin has well-defined anti-inflammatory properties. Some studies showed that the anti-inflammatory effect of curcumin via inhibition on the toll-like receptor (TLR-4), phosphatidylinositol-3 kinase (PI3K), nuclear factor-kappa B (NF-*κ*B), and others resulted in decreased production of IL-6, tumour necrosis factor (TNF-*α*), and interleukin (IL-1*β*), which are proinflammatory cytokines in both *in vitro* and *in vivo* studies [48, 49].

Another study had contradictory results and showed that curcumin had an immunostimulatory effect by increasing IL-6 and TNF-*α* [48, 50]. An additional study had different results that showed curcumin could be used as an immunomodulator for both immunostimulation and immunosuppression [50]. Taken altogether, we can conclude that the results of curcumin administration are inconsistent since it is an immunomodulator that can increase or decrease proinflammatory cytokines.

This evidence raises concerns about the administration of curcumin as a treatment for COVID-19. Some patients with COVID-19 show a hyperinflammatory and hypercytokinaemia known as CRS or a cytokine storm. Administration of curcumin may increase the production of proinflammatory cytokines that may worsen the condition of COVID-19 patients with a cytokine storm. A further and more detailed investigation is urgently needed to clarify the effect of curcumin in the treatment and prevention of COVID-19.

### 4.3. *Cinchona* sp

Cinchona trees *(Chincona L., Raiatea*) from the Andean mountain forests have valuable benefits as a particular component of the trees contains bioactive compounds that can heal fever. This beneficial effect was first discovered by Jesuit missionaries and gradually expanded throughout the world [51]. The bark of the trees produces quinine alkaloids, which were also an effective treatment of malaria for more than several centuries. Quinine has a mode of action that is similar to that of chloroquine, a synthetic antimalaria agent to treat malaria. Thus, it is known as a chloroquine analogue [52].

Nowadays, quinine sulphate has become one of the most wanted drugs in the society for COVID-19 treatment. Inappropriate statements had been made by state officials and doctors that caused public panic. So, people looked for quinine-containing drugs competitively. The behaviour of the people was triggered by a spontaneous reaction because of the high incidence and mortality rate of COVID-19 worldwide. This section will explain the potential of quinine to act as an antiviral agent and function as an immunomodulator in a disease caused by a virus. Further explanation will also discuss the potentially harmful effects of quinine in individuals with or without COVID-19.

Quinine became popular because its effect was similar to chloroquine, an antimalarial drug. Eventually, there were shifts from malarial drug use as antimalarial drugs to their use as potent inhibitors for viral infection. A large amount of data suggested that several antimalarial drugs had been investigated and then concluded that they had some advantages against viral infection [53]. For example, chloroquine showed an antiviral effect against the SARS-CoV infection [54]. A clinical trial proved that hydroxychloroquine improves the SARS-CoV-2 viral load in COVID-19 patients when combined with azithromycin. From this evidence, quinine as a chloroquine analogue might be predicted to have beneficial effects in eliminating the COVID-19 [28].

The first investigation of the quinine effect as an anti-influenza virus infection was conducted by Seeler et al. [55]. Moreover, the antiviral effect of quinine was described by Baroni et al. in their report that evaluated quinine sulphate on HSV-1 HaCat cells infected, which suggested that quinine acts to inhibit the viral infection through indirect pathways, such as activating the protein heat shock response, interfering with multiple pathways during virus replication, and inhibiting NF-kB by blocking the expression of the gene [56].

**Figure 4:**
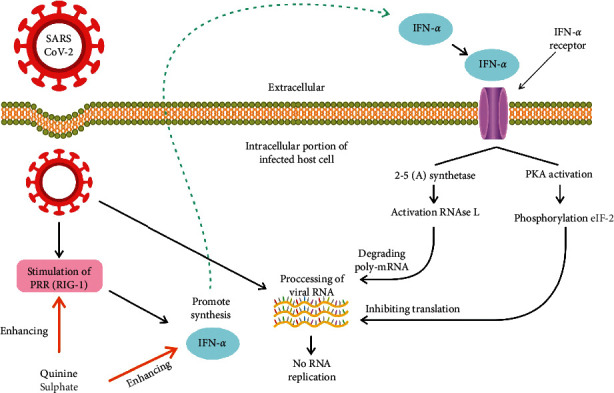
Quinine sulphate has activity as an antiviral agent by enhancing the synthesis of RIG-I and IFN-alpha synthesis. Then, both will block the translation of viral mRNA through the activation of PKR and degrade the viral poly mRNA by activating RNAse (L) Therefore, no viral protein is synthesised.

A recent study invariably mentioned that the proposed antiviral mechanism of quinine is indirectly killing off viruses. Previous research investigated the effect of quinine sulphate on dengue virus-infected cells. As the relative similarity of dengue virus and SARS-CoV-2 structures, it may be possible that SARS-CoV-2 uses some relative methods to infect the cell and trigger cytokines to combating the virus (Figure 4) [57]. The host cells infected by viruses will initiate the release of viral RNA and interfere with normal protein synthesis. However, an expression of a pathogen recognition receptor (PRR) known as RIG-I in the infected host cell increases minimally to promote the IFN-I signalling pathway rather than to elevate gene expression of IFN-stimulated genes (RNase L, PKR) that inhibit the synthesis of the protein, thereby blocking viral replication [58]. The RNase L pathway can eliminate ssRNA in virus-infected cells, while PKR blocks the translation and affect signal transduction [59]. The target of quinine action is the inhibition of genome replication and translation of infected host cells and increased expression of RIG-I and IFN-*α*. It has been proven that IFN-*α* is the cytokine secreted by host cells to fight the viruses [57].

CSS mentioned earlier does not seem to appear after quinine administration in COVID-19 since the available data show that the release of TNF-*α*, the most important cytokine in determining the severity of COVID-19 symptoms, is inhibited by quinine [60, 61]. Because quinine blocks the expression of TNF-*α* at the mRNA transcription level, analysed by northern blot, it will alleviate an inflammatory reaction in microbe-infected individuals rather than promoting the process of inflammation [60].

Although the extract of the cinchona tree might not directly lead to CSS, a systematic review reported by Liles et al. showed that there is a couple of general adverse reactions caused by quinine, such as immune-mediated reactions and toxic reactions. The documented damage involves organ systems including haematologic manifestations: thrombocytopenia and haemolytic anaemia; dermatologic manifestations: photosensitive eczema; systemic reactions: hypotension and anaphylaxis; cardiac manifestations: chest pain and pericarditis; liver toxicity: elevated serum transaminase; kidney manifestations: thrombotic microangiopathy, nephritis, and acute kidney injury [62].

Quinine is rarely categorised as an immunomodulatory agent, but it has immunostimulant and immunosuppressant activities against viral infections. When quinine effectively intensifies the production of the well-known cytokine IFN-*α*, it functions as an immunostimulator to inhibit viruses. Otherwise, quinine inhibits the release of TNF-*α* and has an immunosuppressant effect. These two different activities may have a beneficial effect on people who are infected with COVID-19. However, routine consumption of this herbal medicine by healthy people to prevent COVID-19 is not recommended because of the possibility that it may lead to various harmful events.

### 4.4. Xanthorrhizol

Java turmeric or *Curcuma xanthorrhiza* Roxb (*C. xanthorrhiza*) is an herbal plant that is widely used in Southeast Asian countries. This plant belongs to the *Zingiberaceae* and *Curcuma* genus. Java turmeric originates from Indonesia and has been spread and grown wild in Thailand, Philippines, Sri Lanka, and Malaysia. It also has been used as a food additive to enhance the flavour of food [63]. In addition, this plant has been utilised in the medicine world, and its benefits have been scientifically proven. This plant is used to treat some diseases and as a supplement [49, 63–65].

*C. xanthorrhiza* is widely used as a medication and supplement for specific diseases. This plant has some effects as an antimicrobial, anti-inflammatory, antioxidant, antihyperglycemic, antihypertensive, antiplatelet, nephroprotective, anticancer, and supplemental agent for systemic lupus erythematosus (SLE) [51–54]. *C. xanthorrhiza* is a dried rhizome and contains curcuminoids (1%–2%), volatile oil (3%–12%), xanthorrhizol (44.5%), and camphor (1.39%). Curcumin, monodemethoxycurcumin, and bisdemethoxycurcumin belong to curcuminoids. The presence of xanthorrhizol is specific and can distinguish this plant from *C. longa* [56, 57].

The most common compound in java turmeric is xanthorrhizol. The study about xanthorrhizol active compounds suggested that this compound could decrease inflammatory genes in adipose tissue, liver, and muscle in patients with diabetes mellitus. This study showed that xanthorrhizol treatment inhibited inflammatory cytokine production in adipose tissue and tumour necrosis factor (TNF-*α*) expression. The results showed that xanthorrhizol prevents the recruitment of immune cells to adipose tissue to downregulate the inflammatory cytokine gene. In the liver, xanthorrhizol inhibited CRP, a nonspecific marker of systemic inflammation. Xanthorrhizol also reduced interleukin (IL-1*β*) gene expression in muscle [65]. Another study suggested that xanthorrhizol can decrease the serum level of IL-6 and increase serum transforming growth factor (TGF-*β*) in patients with SLE with hypovitamin D [64]. Taken together, xanthorrhizol can inhibit proinflammatory cytokines and promote the production of anti-inflammatory cytokines.

A study about the anti-inflammatory and antioxidant effects of xanthorrhizol in hippocampal neurons and primary culture microglia was in line with the previous study in patients with diabetes mellitus and SLE. It showed that xanthorrhizol could suppress the generation and secretion of proinflammatory cytokines, such as IL-6 and TNF-*α*. This inhibition process is the result of inhibited inducible nitric oxide synthase (iNOS) and decreased production of nitric oxide (NO) [66–68]. Taken together, xanthorrhizol as an immunomodulatory is an immunosuppressant.

Xanthorrhizol is an immunosuppressant that may be used as a treatment for COVID-19 because of its ability to inhibit proinflammatory cytokines. Patients with COVID-19 are susceptible to CRS. So, the use of xanthorrhizol may lower the proinflammatory response in a patient with COVID-19 with or without CRS. However, the administration of xanthorrhizol needs to be done carefully and with consideration because at present, no study has been conducted with xanthorrhizol in COVID-19. There is still a chance that the administration of xanthorrhizol may worsen the conditions of patients with COVID-19. Using xanthorrhizol for treatment and prevention in COVID-19 still requires more evaluation, especially in the clinical trial setting.

## 5. Conclusion and Recommendation

Based on the previous explanation, we conclude that these herbal medicines might have the capabilities to regulate the production and release of proinflammatory cytokines, interfere with the development of the virus in host cells, and modify certain molecular pathways related to the RAA system. Herbal agents might be useful as treatments to fight COVID-19. Finally, a suggestion for the patients is that it is still not recommended to use the supplementation containing one of these compounds to prevent COVID-19 or to heal the disease without the specific advice or under the direct supervision of a clinician. A suggestion for the clinician is that administration of these herbal medicines must be given carefully to patients, even if they are healthy. This is because various inconsistent data have been reported about these agents. Thus, there is a possibility that these treatments might be associated with the induction of harmful effects. In addition, preclinical and clinical trial evaluations of these herbal agents for COVID-19 have not specifically been conducted, so further investigations related to this are warranted.

## Data Availability

The data supporting this review article are from previously reported studies and datasets, which have been cited.
